# Neurochemical Mechanisms in Excoriation Disorder: Evidence for Therapeutic Interventions

**DOI:** 10.1192/j.eurpsy.2026.11620

**Published:** 2026-07-31

**Authors:** J. A. Castro, F. F. Morais, E. S. Reis, V. K. R. Lemos

**Affiliations:** 1 FESAR (Faculty of Higher Education of the United Amazon), Redenção; 2 FESAR (Faculty of Higher Education of the United Amazon), Redencao; 3 FACIMPA AFYA, Maraba, Brazil

## Abstract

**Introduction:**

Excoriation disorder (skin-picking disorder) is an obsessive-compulsive related condition characterized by repetitive skin-picking behaviors, causing distress and functional impairment. Neurobiological evidence implicates dysregulation in the serotonergic, dopaminergic, and GABAergic systems, affecting impulsivity, inhibitory control, and emotional regulation. Understanding these mechanisms is essential to guide pharmacological and behavioral interventions.

**Objectives:**

This study aims to synthesize and analyze neurochemical evidence in excoriation disorder, evaluating interactions among serotonergic, dopaminergic, and GABAergic systems and their association with frequency, intensity, and severity of compulsive behaviors. Additionally, it seeks to identify therapeutic implications, considering pharmacological (SSRIs, low-dose antipsychotics, benzodiazepines) and behavioral interventions, to inform evidence-based clinical strategies.

**Methods:**

A systematic literature review was conducted covering publications from 2015 to 2025 in PubMed, Scopus, PsycINFO, and UpToDate. Studies included adults diagnosed according to DSM-5 or ICD-10 and addressed neurochemistry, neuroimaging, or neurophysiology of serotonergic, dopaminergic, and GABAergic systems. Original articles, systematic reviews, and meta-analyses in English, Spanish, or Portuguese were considered. Excluded were pediatric studies, small case reports (<5 participants), studies without clear neurotransmission data, and duplicates. Data extracted included neurochemical alterations, correlations with severity and frequency of behaviors, and therapeutic implications.

**Results:**

Evidence indicates dopaminergic hyperactivity in fronto-striatal circuits, linked to compulsive behaviors and reward-seeking. Serotonergic dysfunction, particularly in prefrontal cortex and anterior cingulate, correlates with impaired inhibitory control and emotional regulation. GABAergic deficits contribute to decreased impulse control, perpetuating skin-picking episodes. Patients with greater dopaminergic and GABAergic imbalance show more frequent and severe behaviors, while serotonergic modulation is associated with improved impulse control and anxiety reduction. These findings support combined interventions, integrating SSRIs, low-dose antipsychotics, and habit reversal training (HRT).

**Conclusions:**

Excoriation disorder involves dysregulation of serotonergic and dopaminergic systems, combined with GABAergic deficits, sustaining compulsive behaviors and impairing inhibitory control and emotional regulation. Integrated pharmacological and behavioral strategies can restore neurochemical balance and reduce compulsive episodes. Future studies should explore individualized, multimodal approaches to optimize clinical management and minimize functional impact.

**Disclosure of Interest:**

None Declared

